# Supplementary material for: Effects of substrate and water depth of a eutrophic pond on the physiological status of a submerged plant, Vallisneria natans
Source: PeerJ. 2020 Nov 9;8:e10273. doi: 10.7717/peerj.10273 (PMC7659635; doi:10.7717/peerj.10273)
Supplement: Supplemental Information 1 — Significance of effects are shown by asterisks (***: p < 0.001, **: p < 0.01, *: p < 0.05, +: p <0.1) [file peerj-08-10273-s001.docx]

|  | Mean | | | | | | | | |  | ANOVA results | | | | |
| --- | --- | --- | --- | --- | --- | --- | --- | --- | --- | --- | --- | --- | --- | --- | --- |
|  | Experiment day | | | | |  | Depth (m) | | |  | Time | |  | Depth | |
|  | 0 | 5 | 10 | 20 | 30 |  | 0.5 | 1.2 | 2 |  | *F* | *p* |  | *F* | *p* |
| Temperature (°C) | 21.7 | 25.0 | 23.7 | 23.8 | 23.6 |  | 23.59 | 23.57 | 23.55 |  | 8275 | ^***^ |  | 2.87 |  |
| pH | 8.24 | 8.09 | 8.04 | 8.09 | 7.90 |  | 8.12 | 8.06 | 8.05 |  | 4.55 | ^*^ |  | 0.84 |  |
| DO (mg/L) | 9.96 | 8.59 | 7.54 | 6.39 | 8.25 |  | 8.29 | 8.11 | 8.04 |  | 97.2 | ^***^ |  | 1.47 |  |
| ORP (mV) | 233 | 128 | 68 | 239 | 277 |  | 184 | 189 | 193 |  | 293 | ^***^ |  | 1.28 |  |
| EC (μS/cm) | 446 | 443 | 450 | 430 | 463 |  | 446 | 446 | 447 |  | 263 | ^***^ |  | 0.91 |  |
| Chl-a (μg/L) | 66.4 | 43.2 | 62.0 | 28.7 | 54.9 |  | 49.1 | 51.5 | 52.5 |  | 38.6 | ^***^ |  | 0.81 |  |
| Turbidity (NTU) | 47.3 | 62.0 | 74.7 | 36.2 | 43.5 |  | 51.3 | 52.6 | 54.3 |  | 194 | ^***^ |  | 3.13 | ^+^ |
|  |  |  |  |  |  |  |  |  |  |  |  |  |  |  |  |
| Transparency (m) | 0.25 | 0.27 | 0.27 | 0.28 | 0.30 |  |  |  |  |  |  |  |  |  |  |
|  |  |  |  |  |  |  |  |  |  |  |  |  |  |  |  |
| Light quantum (μmol/m^2^s) | 39.9 | 55.9 | 48.5 | 30.2 | 45.7 |  | 79.3 | 38.3 | 6.7 |  | 1.95 | ^*^ |  | 283 | ^***^ |
|  |  |  |  |  |  |  |  |  |  |  |  |  |  |  |  |
| COD (mg/L) | 37.7 | 35.0 | 75.7 | 26.7 | 28.0 |  | 41.2 | 40.2 | 40.4 |  | 86.0 | ^***^ |  | 0.10 |  |
| TN (mg/L) | 8.50 | 8.48 | 8.23 | 6.05 | 6.96 |  | 8.00 | 7.44 | 7.50 |  | 49.7 | ^***^ |  | 6.52 | ^*^ |
| TP (mg/L) | 0.40 | 0.42 | 0.54 | 0.44 | 0.42 |  | 0.45 | 0.43 | 0.45 |  | 3.82 | ^+^ |  | 0.34 |  |
| NO_3_^-^-N (mg/L) | 5.40 | 5.94 | 4.87 | 4.80 | 5.32 |  | 5.36 | 5.12 | 5.32 |  | 7.88 | ^**^ |  | 1.05 |  |
| NO_2_^-^-N (mg/L) | 0.42 | 0.42 | 0.52 | 0.54 | 0.71 |  | 0.52 | 0.53 | 0.52 |  | 125 | ^***^ |  | 0.17 |  |
| NH_4_^+^-N (mg/L) | 0.36 | 0.96 | 0.06 | 0.09 | 0.61 |  | 0.39 | 0.44 | 0.42 |  | 95.5 | ^***^ |  | 0.68 |  |
