# Supplementary figures and images for: Effects of substrate and water depth of a eutrophic pond on the physiological status of a submerged plant, Vallisneria natans
Source: PeerJ. 2020 Nov 9;8:e10273. doi: 10.7717/peerj.10273 (PMC7659635; doi:10.7717/peerj.10273)

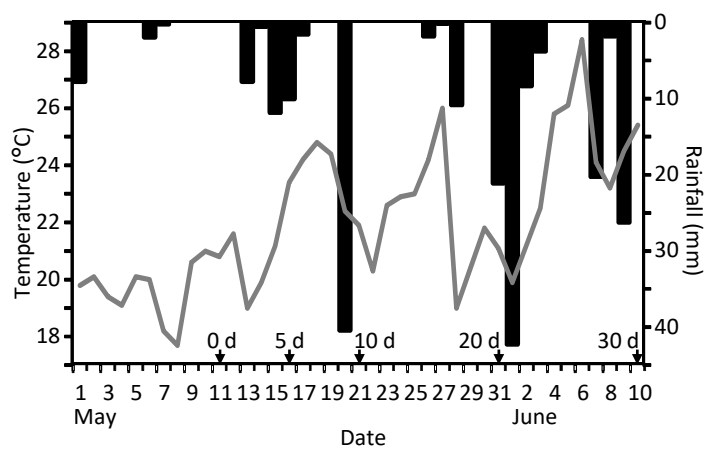

Supplement: Supplemental Information 2 [file peerj-08-10273-s002.pdf]
